# Cultural and Linguistic adaptation and Validation of the World Health Organization iSupport Training Manual for Informal Caregivers of People Living with Dementia to the Yoruba language

**DOI:** 10.1002/alz70860_102788

**Published:** 2025-12-23

**Authors:** Lawrence A Adebusoye, Abiola Obadare, Eniola O Cadmus, Oluwafemi O Olowokere, Mofoluwake Majekodunmi, Olufisayo Oluyinka Elugbadebo, Abimbola O Atanda, Taiwo O Adebowale, Victor Ogaji, Olusegun O Olawunni, Adesola Ogunniyi

**Affiliations:** ^1^ Chief Tony Anenih Geriatric Centre, University College Hospital, Ibadan, Ibadan, Oyo State, Nigeria; ^2^ Chief Tony Anenih Geriatric Centre, University College Hospital, Ibadan, Oyo, Nigeria; ^3^ University of Ibadan and University College Hospital, Ibadan, Oyo, Nigeria; ^4^ University College Hospital, Ibadan, Nigeria; ^5^ College of Medicine, University of Ibadan, Ibadan, Nigeria; ^6^ Institute for Advanced Medical Research and Training, College of Medicine, University of Ibadan, Ibadan, Oyo, Nigeria; ^7^ College of Medicine, University of Ibadan, Ibadan, Oyo, Nigeria; ^8^ Africa Dementia Consortium, Ibadan, Ibadan, Nigeria; ^9^ Institute for Advanced Medical Research and Training, College of Medicine, University of Ibadan, Ibadan, Nigeria

## Abstract

**Background:**

Care for People with Dementia (PwD) is predominantly provided through informal systems, which are often demanding and place a significant burden on caregivers. To enhance the quality of care for PwDs, it is crucial to provide training and support for these informal caregivers. This study documents the process of the cultural adaptation of the World Health Organisation iSupport training programme for informal carers of PwD, emphasizing the participatory strategies used to develop a culturally tailored Yoruba version of the program.

**Method:**

The study took place in University College Hospital, Ibadan, the Yoruba‐speaking part of southwest Nigeria. It was done in four phases: content translation, linguistic and cultural revision by the community advisory board, validation through Key Informant Interviews (KIIs) with formal caregivers, and refinement and final adaptation by experts in the care of PwDs

**Result:**

The reports of each phase of the cultural adaptation process show valuable insights into the development of the Yoruba version of iSupport. Participants agreed that the iSupport manual is comprehensible and easy to understand, with some reporting the need to adapt most scientific terms or phrases and numbers to Yoruba. Some original names were retained as ‘loanwords’ those with Yoruba tones. The KIIs illustrate the challenges faced in caring for PwDs.

**Conclusion:**

The cultural adaptation of the Yoruba version of iSupport has shown the need for culturally sensitive, community‐participatory and comprehensive interventions that address the multifaceted challenges of dementia caregiving in diverse settings. This attempt provides a strong foundation for further research and implementation efforts in LMICs.

